# Monitoring and Surveillance of COVID-19 Survival and Stay Characteristics: A Need for Hospital Preparedness in India

**DOI:** 10.1017/dmp.2020.251

**Published:** 2020-07-15

**Authors:** Ajit Deo Burma, Vinayak Mishra, Sumit Kumar Das, Mohana Balan Parivallal, Senthil Amudhan, Girish N. Rao

**Affiliations:** Department of Epidemiology, National Institute of Mental Health and Neuro Sciences, Bangalore, India; Department of Biostatistics, National Institute of Mental Health And Neuro Sciences, Bangalore, India

**Keywords:** communicable diseases, community health planning, hospital bed capacity, hospital records

As coronavirus disease (COVID-19) is unfolding rapidly with a huge surge after the relaxation of lockdown measures, hospital preparedness becomes crucial for the management of the COVID-19 pandemic in India. For a country like India where 17% of the global population resides in 2.4% of the total global land area, focusing on the descriptive statistics of COVID-19 (number of cases, deaths, and recoveries) alone will prove insufficient for planning the health-services. There is a need to move beyond the descriptive statistics and understand the survival and stay pattern of COVID-19 cases to guide policy-makers to allocate resources and plan contingencies.

In a recent study from India, Kaplan–Meier analysis showed an overall survival rate of 95.7% at day 7 and 95.5% at day 14 of admission.^1^ With no significant difference in survival rate, a cost-effective and comparative analysis on the care provided between 0‐7 and 7‐14 days of admission would have provided valuable insights on the need for hospital care after day 7 of admission. This would have great implications on the guidelines for discharge criteria, home-quarantining, and follow-up, especially when there is a huge surge in COVID-19 cases during the pandemic crisis. Contrasting with current reporting, the recent study from India reported a high recovery rate of 94.9% as it did not include all fresh cases (which are yet to have definite outcomes) in the denominator.^1,2^ We firmly assert that this strategy of recovery-rate estimation (by fixing the confirmed cases at 1 time point with subsequent follow-up till their definitive outcomes) would provide a valid estimate of COVID-19 recovery rate in India.

Unlike the hospitalization rate that can be modeled, estimating length of stay (LOS) requires observation and reporting of individually hospitalized patients.^3^ It can be noted that patient characteristics like severity and so forth had influenced the LOS and those with a severe disease had a shorter LOS.^1^ This may imply that severely affected patients follow an acute course of treatment with a short preclinical phase (from disease onset to symptoms) or possibly a delay in seeking appropriate care.^4^ Though the former is non-modifiable relating to the nature of the disease, the latter can be prevented by timely testing, contact-tracing and risk-profiling. Though challenging, this will be crucial for the preservation of the intensive care unit (ICU) capacity. Given the inherent difficulty and absence of data, most studies from India did not report on the LOS in the ICU to provide better insights on the intensive care needs of COVID-19 patients.

As the COVID-19 pandemic is progressing, the stay and survival characteristics may change because of change in disease characteristics or change in admission/discharge criteria. Ideally, the requirement for hospital resources is planned based on data from the local setting because the age structure, COVID-19 hotspots, and hospital beds are not uniform across the country.^5^ LOS is often not recorded in local reporting or monitoring. Despite its challenges, at least information on LOS by type of care (general ward, dependency unit, and ICU) would make hospital preparedness planning more realistic/pragmatic. This also underscores the need for strengthening the surveillance and monitoring of COVID-19 survival and stay characteristics at various levels of care (primary, secondary, tertiary) to provide timely inputs for a dynamic and iterative hospital preparedness plan at the state and district levels. This will ensure timely redistribution of resources for optimal COVID-19 care in the community – definitely a need of the hour.
